# Load-Bearing Capacity of Incisors Restored Using Fiber-Reinforced Composite Post-Core Systems

**DOI:** 10.3390/dj13030125

**Published:** 2025-03-13

**Authors:** Keiichiro Uchikura, Sufyan Garoushi, Kohji Nagata, Pekka K. Vallittu, Noriyuki Wakabayashi, Lippo Lassila

**Affiliations:** 1 Advanced Prosthodontics, Oral Health Sciences, Graduate School of Medical and Dental Sciences, Tokyo Medical and Dental University, Tokyo 113-8510, Japan; keiichiro.uchikura@utu.fi (K.U.); k.nagata.rpro@tmd.ac.jp (K.N.); wakabayashi.rpro@tmd.ac.jp (N.W.); 2 Department of Biomaterials Science and Turku Clinical Biomaterial Center, Institute of Dentistry, University of Turku, 20014 Turku, Finland; pekval@utu.fi (P.K.V.); liplas@utu.fi (L.L.); 3 Asclapia Medical and Dental Institute, Tokyo 156-8506, Japan; 4 Wellbeing Services County of Southwest Finland, 20521 Turku, Finland

**Keywords:** incisor, fiber composite, endodontic post, fracture resistance

## Abstract

**Objectives:** This study aimed to analyze the load-bearing performance of upper incisors and evaluate the curing of the luting polymer composite at various depths within the canal. **Methods:** A total of one hundred maxillary central incisors (10 groups, *n* = 10/group) were subjected to various restorative techniques. Approach A used Gradia Core for post-core and crown; Approach B employed prefabricated fiber posts (4 mm or 8 mm) with Gradia for luting and core build-up; Approach C used short-fiber composite (everX Flow) for post-core build-up; and Approach D used fiber posts with everX Flow for luting and core build-up. Restorations underwent cyclic fatigue (40,000 cycles at 95 N) and quasi-static fracture testing. Surface hardness of luting polymer composites was also measured. **Results:** Data showed that restorations with additional fiber posts (Approaches B and D) had significantly higher load-bearing capacity (*p* < 0.05), while post material and length had no significant impact (*p* > 0.05). Short-fiber composite as luting and core material (Approach D) enhanced load-bearing performance compared to Gradia-based restorations (Approach B, *p* < 0.05). **Conclusions:** The use of short-fiber composite as both the post luting and core material in restoring compromised incisors, along with a conventional fiber post, demonstrated favorable results in terms of load-bearing capacity.

## 1. Introduction

Typically, root canal-treated (RCT) teeth frequently require substantial reinforcement through various post-core system applications. Post selection depends on the amount of intact coronal and internal root structure, as well as the tooth’s type and location within the dental arch. The principal aim of using a post is to improve retention in cases where the existing tooth structure is insufficient to support the core restoration [1,2]. Post placement is recommended in anterior teeth when the remaining crown structure is <50% [3,4]. An essential determinant influencing the success of post restorations is the existence or nonexistence of a coronal dentin height measuring 1.5–2 mm, commonly referred to as the “ferrule” after the preparation. The ferrule aims to redistribute stress experienced by the outer coronal third of the root, thereby modifying the fracture pattern to one amenable to restoration [5].

Different categories of fiber posts have emerged to provide dentists with an alternative to casts or ready-made metal posts for RCT teeth restoration. These fiber posts provide a closer match in terms of the modulus of elasticity to dentin than metal posts [6]. This similarity is clinically significant, as it allows for a more uniform distribution of stress along the root, reducing the risk of root fractures [6]. It is noteworthy that the front teeth that have been restored using prefabricated posts demonstrate a fracture rate that is three times greater than that observed in posterior teeth [7]. This is partly attributed to the elevated horizontal forces to which the front teeth are subjected due to their location within the dental arch. Demands are placed on the interface between the post and root canal when greater horizontal forces are at play, and any potential imperfection may eventually lead to failure.

The most prominently observed failures involve either failure in post retention or post fracture, in the case of fiber posts [8]. These issues are attributed to several factors, such as the post material’s inability to form an efficient bond with the luting or core build-up material, the irregular shape and cross-sectional profile of the root canal, or the perceived weakening of the root structure during post space preparation. Studies in the literature indicate that the quantity and orientation of the fiber post, particularly in the critical cervical region of the tooth, play a key role in determining the effectiveness of restorative procedures involving post placement [9]. Improperly fitted posts, especially at the coronal level, result in a thicker layer of luting resin. This condition improves the formation of voids and cracks under loading, thereby increasing the risk of post debonding.

One approach to addressing this issue is to design a customized fiber post using multiple unidirectional pre-impregnated fiber bundles [10,11]. This method provides better adaptation to large, irregular root cavities compared to a single, prefabricated, centrally positioned post, while also minimizing the amount of luting cement required. Alternatively, another method is to directly use short fiber-reinforced composites (SFRCs) inside the root canal to construct the post and core [9,12,13,14,15]. This “monoblock” technique entails filling both the root canal space and the coronal cavity with SFRC in horizontal increments of 4–5 mm thickness [14,16]. A flowable version of SFRC was released in 2019 that has the benefit of being easily adaptable in small areas, such as root canals. This raises concerns regarding the use of this flowable SFRC for post-core build-up or traditional fiber post cementation in restoring the anterior teeth after root canal therapy and in the presence of a ferrule. To the best of our knowledge, the available literature has not extensively investigated this aspect. However, despite its promising mechanical properties, parameters such as film thickness, adaptation, microleakage because of shrinkage stress, void formation, and bonding strength to dentin and fiber posts are all concerns that need to be further investigated.

Many in vitro studies, including those that utilize Finite Element Analysis (FEA), have played a significant role in evaluating the mechanical behavior of anterior restorations, with or without posts and ferrules [17,18]. Some studies have reported that the use of posts did not improve the fatigue resistance of anterior teeth with or without a ferrule [19,20,21]. In contrast, other studies have shown better mechanical performance in teeth restored with posts [22,23,24]. The inconsistency in the literature regarding the influence of posts on restoration of RCT anterior teeth has led to the exploration of alternative approaches.

Unlike previous studies, this research study specifically evaluates the load-bearing performance of SFRC used as both the post and core material. These findings provide a novel perspective on the biomechanical reinforcement of compromised anterior teeth, offering valuable insights for improving clinical outcomes in restorative dentistry. The null hypothesis proposed is that anterior teeth restored using SFRC as a post-core would demonstrate similar fracture load values to those restored with conventional restorative techniques.

## 2. Materials and Methods

**Figure 1:**
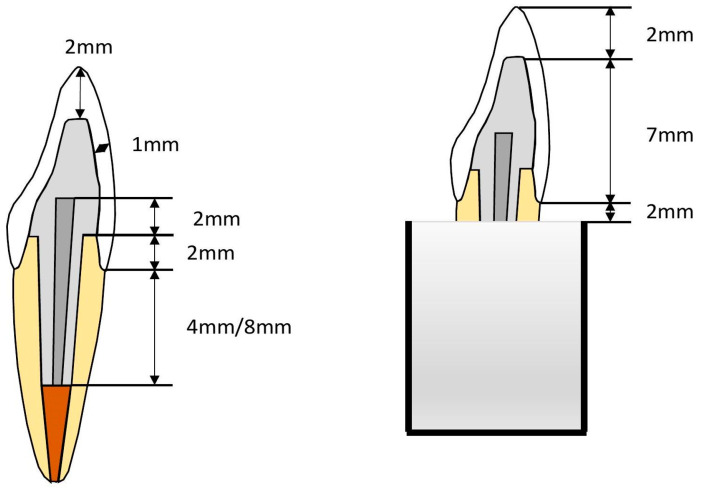
Illustration of post space preparation for restored maxillary incisors.

**Figure 2:**
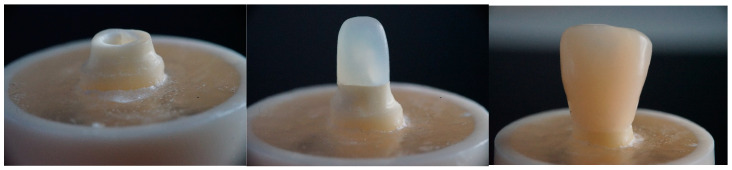
Steps of the post and core fabrication.

**Figure 3:**
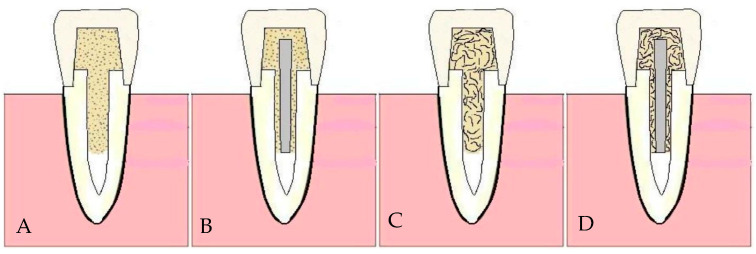
Schematic figure representing the final restorative approaches (**A**–**D**) with different post & luting/core foundations. A transparent template matrix was used to ensure uniform crown contours among specimens.

Table 1 provides comprehensive details of all the materials used in this investigation. A total of 100 fully intact upper central incisors, characterized by uniform mesiodistal and buccolingual dimensions and a root length of 14 mm, were selected. This study included only teeth deviating by a maximum of 10% from the calculated mean to ensure measurement consistency. A 10% deviation threshold was selected based on established methodologies in similar studies to ensure consistency in root dimensions while maintaining a representative sample of natural variations [14]. This limit value helps reduce outliers that may affect the mechanical performance of restorations. The crowns of these teeth were horizontally sectioned 2 mm above the cementoenamel junction (CEJ) using a diamond disk with water cooling. A single operator (KU) performed all tooth preparations. Post space preparations were performed using post drills (Parapost stainless drills, Coltène/Whaledent, Mahwah, NJ, USA) via a low-speed handpiece with water cooling after removing and cleaning the pulp and periodontal tissues. Subsequently, the teeth were affixed just 2 mm below the CEJ on an acrylic block with a 2.5-cm diameter using autopolymerized acrylic resin (Palapress; Heraus Kulzer, Wehrheim, Germany) (Figure 1). The next steps involved post-core and crown fabrication based on four distinct restorative approaches (Figure 2 and Figure 3). These approaches were further subdivided into 10 groups, each consisting of 10 specimens, and are detailed in Table 2.

Approach A involved the use of the Gradia Core for both the post and core foundations and the complete crown constructed from conventional particulate-filled composite (PFC) (G-aenial Universal Injectable: control).

Approach B entailed the use of prefabricated fiber posts, either MI or SN, with two different lengths (4 and 8 mm) placed inside the root canal. The Gradia Core served as the post-luting and core build-up material, and the complete crown was constructed from PFC.

Approach C indicated the use of both the fiber post and core designed from everX Flow (SFRC), with the complete crown made of PFC.

Approach D followed the same procedure as approach B but with everX Flow used as the luting and core build-up material.

### 2.1. Core Build-Up and Post Fabrication

The coronal aspect of the teeth underwent etching with a 37% phosphoric acid etching gel (Scotchbond, 3M ESPE, St. Paul, MN, USA) for 20 s. After etching, the teeth were thoroughly rinsed and gently air-dried. Dentin adhesive was applied following the manufacturer’s guidelines (G2-BOND Universal, GC, Tokyo, Japan). A transparent template matrix (EXACLEAR, GC, Tokyo, Japan) was used as a guide during core fabrication to ensure uniform core dimensions. Composite cores, extending 5 mm incisally to the sectioned tooth surfaces, were constructed and polymerized incrementally, with each layer exposed to a polymerization light source (Elipar TM S10, 3M ESPE, Seefeld, Germany) for 20 s per layer. The light source emitted wavelengths of 430–480 nm, with a light intensity of 1.600 mW/cm^2^. Post space preparations were performed using post drills (Parapost stainless drills, Coltène/Whaledent, Mahwah, NJ, USA) with a low-speed handpiece and water cooling.

Approach A involves the creation of both posts and cores using Gradia Core. Posts measuring 4 mm in length were formed by directly applying Gradia Core material to the prepared root canals and polymerizing it in bulk. The Gradia Core material was introduced into the prepared root canals using an applicator tip and polymerized in bulk for 40 s with a light source (Elipar TM S10) positioned at the coronal surface. The polymerization process involved incrementally building the core in 2 mm layers, with each layer cured for 20 s, following the method described by Lassila et al. [1]. Approach B entails fiber post cementing using a dual-cure luting material (Gradia Core). The luting material was prepared following the manufacturer’s instructions and was introduced into the root canal using atomic tips.

Approach C had both posts and cores designed using SFRC. Posts (4 mm in length) were generated by applying SFRC material into the prepared root canals and polymerizing them in bulk. Cores were constructed and polymerized incrementally, with each 2-mm layer exposed to a polymerization light source (Elipar TM S10) for 20 s. Approach D involved cementing the fiber posts with SFRC. Prefabricated glass fiber posts (Ø 1.6 mm), namely MI Core Fiber Post and Snowpost, underwent surface treatment with G2 bond adhesive before being slowly inserted into the luting-filled root canal. Any remaining luting material was eliminated at the sectioning level once the posts reached the intended lengths (4 and 8 mm). The cement was then cured with a light source for a minimum of 40 s (Elipar TM S10), positioned at a 45° angle near the root of the post. The posts extended 2 mm above the coronal surface of the prepared teeth, after which the cores were constructed and polymerized following the aforementioned procedure.

### 2.2. Crown Construction

Crown construction replicated a chair-side direct fabrication approach using flowable light-cured PFC (G-aenial Universal Injectable). A clear template matrix was utilized to replicate the ideal crown contour for fabrication to reduce variability among specimens. The use of a transparent template matrix ensured uniform crown contours, reducing variations in crown shape, thickness, and dimensions among specimens.

A crown template was created and filled with PFC, then pressed and positioned over the built-up core. The crown was subsequently subjected to light curing from the exterior from various angles (20 s each). The light source was located at a close distance (1–2 mm) to the crown surface. The crown template was removed once polymerization was complete. Afterward, all restorations were polished using Komet polishing abrasives (Komet, Rock Hill, SC, USA) to ensure clear demarcation between the root and crown materials (Figure 2). Before testing, all constructed crowns were immersed in water at room temperature for 48 h to ensure that the specimens were adequately hydrated, post-polymerized, and stable.

### 2.3. Testing the Load-Bearing Capacity

All restored teeth (*n* = 100, 10/per group) underwent cyclic fatigue aging before conducting the quasistatic fracture load test (Table 2). Each restored tooth was securely attached to an inclined metal base within an acrylic block, resulting in a 45° angle between the palatal surface and the loading tip (spherical, Ø 2 mm). The inclined metal base was securely mounted onto the fatigue testing device (Cera Test 2K, SD Mechatronik, Feldkirchen, Germany). Following the protocol established in previous studies [14], the crowns underwent 40,000 cycles of mechanical dynamic loading, where the loading tip moved up and down in a controlled oscillating manner under wet conditions (water). The fatigue testing was performed with a maximum force of 95 N at a frequency of 12 Hz. The load was generated by six powerful magnets arranged in a circular pattern interacting with a steel plate. The testing setup included both a load sensor and an optical displacement sensor, with the integrated load cell capable of measuring up to 2 kN.

**Figure 4:**
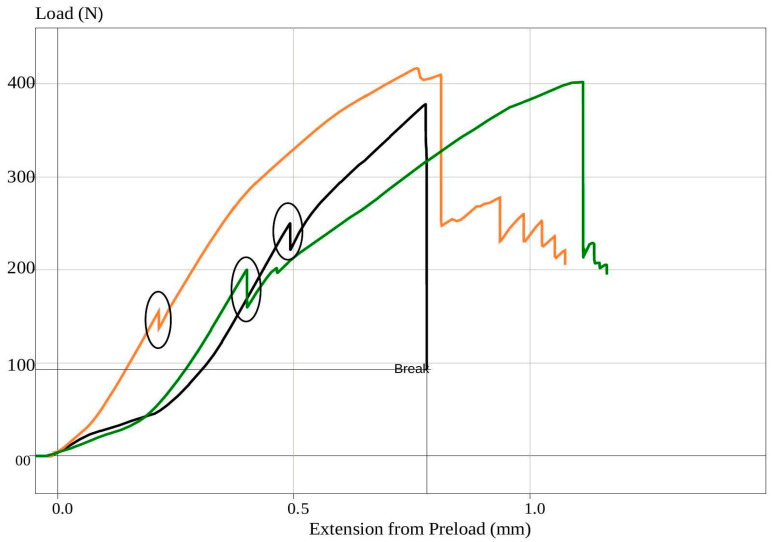
Representative load–displacement curves from different testing groups (Orange: Gradia 8 m MI post; Black: Gradia 8 m SN post; Green: everX 4 m MI post) illustrating the fracture load testing results. Circles highlight the initial drop in the curves, indicating the point of initial failure before the final failure drop.

A quasistatic load was imposed on the restored teeth using a universal testing machine (Lloyd model LRX, Lloyd Instruments Ltd., Fareham, UK), at a rate of 1 mm/min, following cyclic fatigue aging. The initial fracture load was determined from the load–displacement curve (Figure 4), which revealed internal cracks in the structure without visible signs of external damage. The maximum fracture load was then established through visual inspection of the structural damage, in conjunction with the load–displacement curve. The fractures observed in each specimen were visually assessed and categorized into repairable and irreparable types based on consensus among the three examiners. The classification criteria for repairable and irreparable fractures were based on the location of the fracture relative to the cementoenamel junction (CEJ), as described in previous studies [12]. A repairable fracture was characterized by a break that terminated above the CEJ. The fractured tooth can be restored and retained in the mouth in such cases. Conversely, an irreparable fracture extended below the CEJ, indicating that the tooth would likely require extraction.

### 2.4. Surface Hardness Test

Following the loading test, restored incisors (*n* = 3) from each group were vertically sectioned to assess the microhardness inside the root canal using a ceramic cutting disk operating at 100 rpm (Secotom-50, Struers, Copenhagen, Denmark) with water cooling. Subsequently, the sectioned tooth was gently polished using #4000-grit silicon carbide papers at 300 rpm under water cooling, facilitated by an automatic grinding machine (Rotopol-1, Struers, Copenhagen, Denmark). A Vickers indenter (Duramin 40, Struers, Copenhagen, Denmark) equipped with a 40× objective lens, applying a load of 1.96 N for 15 s, was used to measure the surface hardness (VH) of the luting polymer composites (Gradia Core and SFRC) within the canal. Each examined sectioned restoration underwent six indentations at the top (coronal part of the canal) and bottom (apical part, 8 mm) of the canal. The diagonal length impressions were measured, and Vickers values were converted into microhardness values using the following formula:

$$\mathrm{VH}=\frac{1854.4\times P}{d^{2}}$$

where VH represents the Vickers hardness in kg/mm^2^, *P* denotes the load in grams, and *d* indicates the length of the diagonals in micrometers. The constant 1854.4 is used to convert the units from the Vickers test to the appropriate hardness scale (kg/mm^2^), based on the geometry of the diamond indenter used in the test.

### 2.5. Statistical Analysis

Statistical analysis of the dataset involved using a two-way analysis of variance (ANOVA) and subsequent application of the Tukey honestly significant difference test at a significance level (α) of 0.05. This analysis aimed to evaluate the distinctions among the load-bearing capacities of the examined restorations. The Statistical Package for the Social Sciences version 27 (IBM Corp, SPSS, Armonk, NY, USA) was used for this analysis. This analysis indicated the load-bearing capacity as the dependent variable and the type of core material and the presence or absence of a post as the independent variables. One-way ANOVA was also conducted. Additionally, Levene’s test for equality of error variances was executed to evaluate the normal variation in outcomes.

## 3. Results

**Figure 5:**
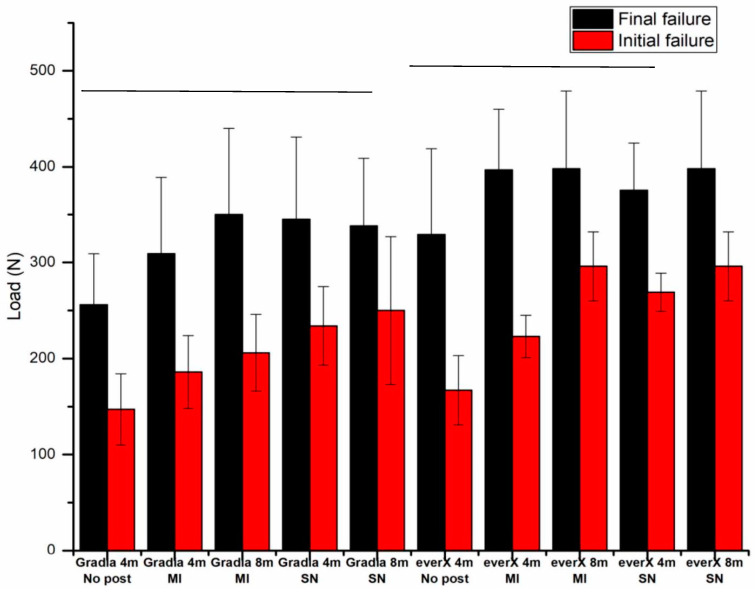
Comparison of load-bearing capacities for the different restorative approaches. Groups (final failure) joined by a horizontal line are not significantly different (*p* > 0.05).

Figure 5 illustrates the load-bearing capacity of teeth restored with different techniques. Statistical analysis using ANOVA revealed that the choice of restoration technique (with or without post and different core materials) demonstrated a substantial effect on the capacity to support loads (*p* < 0.05). However, some interaction between the groups was observed, although it was not statistically significant (*p* > 0.05).

Specimens using SFRC (everX Flow) as both the post cement and core build-up material exhibited significantly greater load-bearing capacities (*p* < 0.05) when compared to groups using Gradia Core (Figure 5). The data further indicated that restorations additionally reinforced with prefabricated fiber posts (approaches B and D) exhibited a higher load-bearing capacity (*p* < 0.05) in contrast to restorations without fiber posts (approaches A and C). However, the difference was not observable based on the type of post used (*p* > 0.05). Additionally, no statistically significant difference (*p* > 0.05) was observed between post lengths of 4 and 8 mm. Concerning fracture patterns, most restored teeth mainly demonstrated an irreparable fracture type (Table 3).

**Figure 6:**
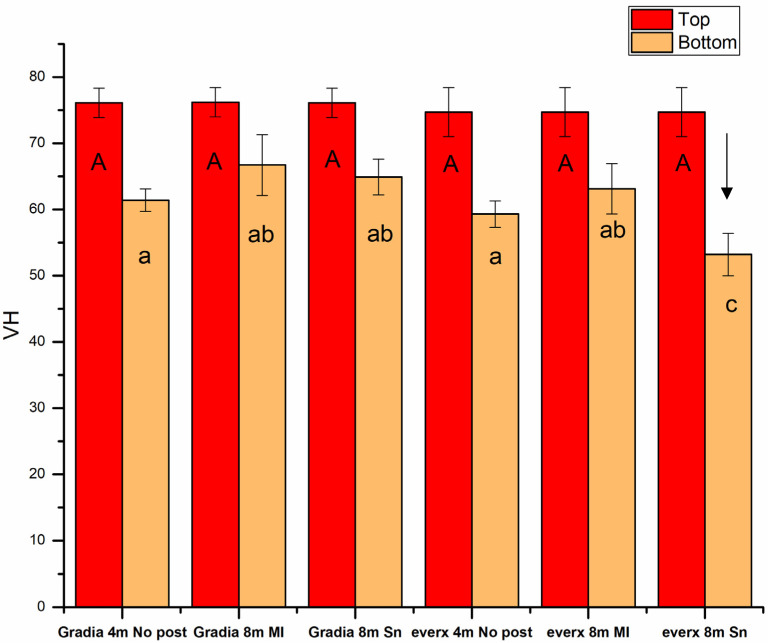
Vickers hardness (VH) values of luting polymer composites measured at different depths in the root canal. The arrow above the column shows that the VH value of this group fell below 80% of the value in the coronal part. The same lowercase letter in the bottom measurements and the uppercase letter in the top measurements indicate non-statistically significant differences (*p* > 0.05).

The VH values of the composite core materials (Gradia Core and SFRC) demonstrated a gradual decrease (*p* < 0.05) as the depth increased from the coronal to the apical part of the canal (Figure 6). No significant difference (*p* > 0.05) in the VH values was observed between the tested core composites at various depths. However, SFRC exhibited a notable decrease (below 80%) in VH values when used with Snowpost at a depth of 8 mm. A significant difference (*p* < 0.05) in the surface hardness of SFRC was found when used with two different posts (MI and Snowpost) at 8 mm depth in the root canal, but no such difference was observed for Gradia Core (Figure 6).

## 4. Discussion

Restoring RCT incisors that have undergone substantial dental structural loss is a key clinical challenge. The choice of an appropriate post-core system is crucial to the treatment’s overall effectiveness [25]. This study used various fiber-reinforced post-core systems to improve the structural integrity of the compromised RCT anterior teeth. Contrary to our initial hypothesis, our results indicate a substantial disparity in fracture behavior among the different restorative techniques used. Therefore, the study hypothesis must be rejected.

Within this sequence of experiments, an effort was made to use flowable SFRC as the core material in constructing direct crown restorations, either with or without adding conventional prefabricated unidirectional glass fiber posts, essentially creating bilayered restorations. Notably, the flowable SFRC used in this study, known as everX Flow, has previously been documented for its remarkable fracture toughness and flexural strength, as stated in previous research [26,27]. To the best of our knowledge, no other dental composites have fracture toughness values of >2.6 MPa m^1/2^. Consequently, we expected that the post-core system, with the support of SFRC, would effectively bear the loads typically encountered in the context of full anterior crown restorations. This study revealed that specimens restored using traditional fiber posts (approaches B and D) demonstrated a noticeably greater capacity to support loads than specimens restored without fiber posts (approaches A and C). The higher load-bearing capacity of approaches B and D is attributed to the reinforcement provided by the prefabricated fiber posts, which distribute occlusal forces more effectively and enhance the structural integrity of the restoration. The aforementioned result contradicts earlier research conducted by Garoushi et al. [12], who revealed no appreciable variation in fracture resistance between anterior decoronate teeth treated with conventional fiber posts and those restored solely with SFRC post and core. However, they used experimental SFRC with a greater fiber aspect ratio. Additionally, our results were in contrast to those of Bijelic et al. [28], although the anterior teeth that were evaluated in their research had sufficient ferrule. In contrast, our findings align with previous studies that emphasize the strengthening effect of fiber posts on restored incisors [10,29]. Notably, other studies have revealed that fiber posts do not support teeth and may increase the risk of fatal failures [30,31].

Posts that are made of glass fiber characterized by a flexural modulus that closely resembles dentin effectively distribute occlusal forces throughout the root structure. The adhesive properties of fiber posts to root dentin play a vital role in their resistance to dislodgement [9]. Hence, establishing a robust adhesive connection between the composite matrix of fiber posts, the luting resins, and the dentin of the root canal is imperative to ensure consistent retention. The prefabricated MI glass fiber posts used in the current investigation exhibit excellent bonding capabilities with luting and core materials due to their semi-interpenetrating polymer network (-IPN) polymer matrix structure, thereby facilitating dependable surface retention [9]. This observation aligns with the results of several researchers who have demonstrated that the potential for resin bonding to penetrate the semi-IPN polymer matrix of fiber posts creates an opportunity for establishing a strong connection between the fiber posts, luting cement, and composite cores [32,33]. Previous research has proposed that the supplementary use of a silane-coupling agent notably improves the adhesion of the luting resin to the post while the Snowpost manufacturer indicated the presence of silanization on the post surface during production [28]. Our study applied a G2 bond adhesive to the fiber surface, which included silane. This application results in robust bond development. These strong bonds resulted from interactions between silanol groups and silica, thereby forming siloxane bonds [34].

Significantly, in our experiments, the load-bearing capacity of approach D, where a SFRC was applied as both the post cement and core build-up material, ranked as the highest among all the experimental groups (Figure 5). This result aligns with previous research, which highlighted that SFRC establish a tight connection with the root canal walls and fiber posts, thereby effectively mitigating the potential drawbacks of using a weaker interface between them. This previous study revealed that the proper placement of short fibers within the canal from a biomechanical perspective helps reduce the potentially damaging tensile stresses that occur when the restoration is subjected to loading [35]. Various studies have revealed that luting resins, specifically in the cervical region, can experience significant stress [9,10,11]. Laboratory fatigue studies have indicated that tiny cracks or microfractures in post luting cement represent the initial mode of failure, thereby facilitating the progression toward catastrophic failure [36,37].

The available literature provides data on the fracture toughness values of different light/dual-cured post luting resins, ranging between 0.5 and 1.3 MPa m^1/2^. These values were inferior to the fracture toughness of flowable SFRC [38,39,40]. The question emerges regarding the potential of light-cured SFRC to attain satisfactory polymerization within the root canal. Generally, the presence of translucency in light-transmitting posts is advantageous for luting procedures because it allows light to pass through the post and reach the inner regions of the root canal; this facilitates the effective polymerization of the luting resin [41]. Our study used two types of fiber posts: MI post, known for its high light transmission (referred to as a light guidance post), and Snowpost, which is a more opaque, esthetic post made of fibers of silica and zirconia embedded in a resin matrix, potentially affecting its light-transmitting properties [41]. However, teeth that were restored using these two types of posts exhibited no distinction in load-bearing capacity.

Earlier research revealed that the flowable SFRC material can undergo effective polymerization within the root canal, even without using a fiber post. However, this polymerization process only marginally attains the microhardness levels achieved by dual-cure materials [16]. This outcome is associated with the translucency of the material and the randomized arrangement of fibers within it, which are capable of conducting and dispersing light over longer distances [41,42,43,44]. This investigation established the highest VH value for each examined composite at the coronal part of the canal (0 mm depth) as the baseline, indicating the optimal degree of conversion for each composite. The ratio of VH at the bottom or apical part of the canal (8 mm depth) to the baseline was considered, with a ratio exceeding 80% frequently used as a minimum acceptable threshold value, in assessing the polymerization capability of each composite [45]. This computed approach indicated that light-cure flowable SFRC is safely used up to a depth of 8 mm inside the canal when employing a fiber post for light guidance (Figure 6). This observation is intriguing and may be ascribed to the light-transmitting capability of the MI fiber post, the presence of short glass fibers within the canal, and the higher transparency noted in the SFRC material. This finding agrees with the latest results of Frater et al., who revealed that light curing via the glass fiber post results in outstanding hardness for the SFRC material in the apical layer in the root canal [45]. However, the light transmission capability varies among different FRC posts, which explains the drop in the VH value observed when Snowpost was used with SFRC material (Figure 6). Le Bell-Rönnlöf et al. revealed that the MI fiber post demonstrated a high amount of light energy transmission through its tip [46]. The existing literature has consistently revealed that transmitted light gradually decreases as it moves apically along the length of the fiber post, and further diminishes as it penetrates deeper into the root canal.

No statistically significant differences in the different post lengths were observed between the groups using 4- or 8-mm post lengths in our investigation (Figure 5). Notably, Cecchin et al. [47] proposed that using a post that extends halfway down the root length enables the effective use of glass fiber posts of intermediate length to impart restoration resistance to fractures. This approach coincides with the concept of minimal intervention, in which a post length extending to a half-length root canal prevents unnecessary canal preparation and safeguards against root fractures. The current investigation supports this by revealing comparable load-bearing values between short and long posts. The use of shorter fiber posts offers advantages in terms of bonding and placement in the context of dental practice. Hatta et al. [48] reported that using a short fiber post system (extending to one-third of the root length) with a greater diameter, matching that of the root canal, results in higher strength than a single long fiber post. However, other studies [49,50] have indicated that two-thirds of the root canal length demonstrates greater fracture resistance than half the canal length. Therefore, further research is required to support the concept of using short FRC posts.

Our investigation identified the initial fracture load by analyzing the load–displacement curve, which marked the appearance of the first internal cracks within the restored incisor. Notably, no visible damage was observed in the tooth structure during this loading phase. Consequently, we determined the initial fracture load by identifying the initial drop in the load–displacement curve (Figure 4). Furthermore, the behavior of the initial fracture load did not exhibit any noticeable distinctions when compared with the behavior of the final or maximum fracture load. It corresponded to a high initial load in instances where a high final fracture load was achieved. Among the various restorative approaches tested, the approach that involved the use of SFRC as both the post cement and core build-up material exhibited the highest average initial fracture load.

The visual analysis of the fracture mode of the restorations revealed that most specimens demonstrated predominantly irreparable fracture patterns. In these cases, a characteristic radial crack extended from the point of load contact over the entire width of the crown/core, extending the remaining tooth structure. This observation contradicts the results from previous studies indicating that anterior restorations featuring a reinforced SFRC core with or without fiber posts tend to display a more reparable type of fracture compared to specimens restored with conventional composite core material [12,35]. The effects of cyclic fatigue aging may play a role in these outcomes. However, none of the restorations demonstrated adhesive failure even under the application of extreme loads. This indicates a high level of bonding at different interfaces, which contributes to the observed results.

In line with other laboratory-based loading experiments, our study applied equal fracture forces (at a 45° loading angle) to the incisor margin, which is acknowledged as the maxillary central incisor’s most crucial mechanical area. The literature indicates that the highest chewing forces on front teeth exhibit some variation, but the common value is approximately 200 N [51]. The load-bearing values obtained in our study, following cyclic fatigue aging, fell within the range of 260–400 N. Thus, all the tested approaches exceeded this typical range. The selected fatigue aging protocol (40,000 cycles, Fmax = 95 N, frequency = 12 Hz) was selected to simulate masticatory forces and short-term fatigue conditions. According to the literature [52,53], our cyclic fatigue aging method is equivalent to only 5 months in vivo (300 inclined loading cycles per day). The chosen protocol aligns with established methodologies in similar studies [14]. The 45° loading angle for quasi-static fracture testing was used to mimic the oblique forces typically exerted on maxillary incisors during mastication. While this approach effectively evaluates short-term performance, it does not consider prolonged fatigue, extended water storage, or thermal aging, all of which may influence long-term outcomes. Future studies should address these limitations.

The use of crowns prepared and tested on teeth embedded in self-cure denture base material with a low elastic modulus could be considered a limitation of this study. Sakaguchi et al. investigated the influence of different substructure materials on the fracture behavior of composite crowns [54]. Their findings indicated that composite crowns mounted on resin models exhibited significantly higher load-bearing values compared to those mounted on metal models. According to their study, the load-bearing capacity of crowns made from brittle materials is influenced by two key factors: the bonding strength at the interfaces and the compatibility of the flexural modulus between the crown and the supporting material. Another limitation of this study is the lack of simulation of the periodontal ligament. The periodontal ligament plays a crucial role in mimicking the natural mobility of the tooth and influencing fracture behavior. Previous studies have shown that excluding the artificial periodontium in loading tests resulted in fracture forces nearly twice as high compared to tests that included the periodontium [55,56,57].

Consequently, further investigations are warranted to corroborate the outcomes of this in vitro study. Root canal treatment (RCT) was not performed in this study to minimize variability and ensure standardized testing conditions. This approach allows a focused evaluation of the mechanical properties of post-core systems without introducing variables related to RCT, such as sealing or preparation differences. Future research could integrate RCT to assess the combined effects on restoration performance.

## 5. Conclusions

Within the limits of the present investigations, the use of flowable SFRC as both the post luting and core material in restoring compromised RCT incisors, alongside a standard fiber post, demonstrated favorable results in terms of load-bearing capacity. However, it is important to note that these findings are based on in vitro conditions. Clinical studies and long-term evaluations are required to validate the results and confirm the applicability of these findings in clinical practice.

## Figures and Tables

**Table 1:** Materials used in the study and their compositions.

Brand (Code)	Manufacturer	Type	Composition
G-aenial Universal Injectable (PFC)	GC Corp, Tokyo, Japan	Conventional particulate composite	Dimethacrylate monomers, Barium glass, silica 69 wt%
everX Flow (everX)	GC Corp, Tokyo, Japan	Flowable fiber-reinforced composite (bulk shade)	Bis-EMA, TEGDMA, UDMA, micrometer-scale glass fiber filler (100–300 µm & Ø 7 μm), Barium glass 70 wt%, 46 vol%
Gradia Core (Gradia)	GC Corp, Tokyo, Japan	Dual-cured core build-up composite	Methacrylic acid ester: 20–30 wt%, fluoro-alumino-silicate glass: 70–75 wt%, silicon dioxide: 1–5 wt%.
MI Core Fiber Post (MI)	GC Corp, Tokyo, Japan	Regular fiber post	UDMA, PMMA, glass fibers
Snowpost (SN)	Abrasive Technology, OH, USA	Regular fiber post	zircon-rich glass fiber embedded in epoxy resin matrix

UDMA: urethane dimethacrylate; TEGDMA: triethylene glycol dimethacrylate; Bis-EMA: ethoxylated bisphenol-A-dimethacrylate; PMMA: polymethylmethacrylate; wt%: weight percentage.

**Table 2:** Different post and luting/core restoration systems.

Group (*n* = 10)	Post & Luting/Core Restoration	Restorative Approach
Gradia 4m No fiber post	Post (4 mm) and core build-up was accomplished using Gradia without any regular fiber post	A
Gradia 4m MI post	Post (4 mm) and core build-up was accomplished using MI post & Gradia	B
Gradia 8m MI post	Post (8 mm) and core build-up was accomplished using MI post & Gradia	B
Gradia 4m SN post	Post (4 mm) and core build-up was accomplished using SN post & Gradia	B
Gradia 8m SN post	Post (8 mm) and core build-up was accomplished using SN post & Gradia	B
everX 4m No fiber post	Post (4 mm) and core build-up was accomplished using everX without any regular fiber post	C
everX 4m MI post	Post (4 mm) and core build-up was accomplished using MI post & everX	D
everX 8m MI post	Post (8 mm) and core build-up was accomplished using MI post & everX	D
everX 4m SN post	Post (4 mm) and core build-up was accomplished using SN post & everX	D
everX 8m SN post	Post (8 mm) and core build-up was accomplished using SN post & everX	D

In Groups A and C, the composite material was directly used to form a post shape within the canal, without the use of a prefabricated post.

**Table 3:** Distribution of various fracture patterns of the tested restorations.

	Only Tooth # (Irreparable)	Tooth and Restoration # (Irreparable)	Only Restoration # (Repairable)
Gradia 4m No post	4	4	2
Gradia 4m MI	6	4	0
Gradia 8m MI	3	5	2
Gradia 4m SN	10	0	0
Gradia 8m SN	7	3	0
everX 4m No post	5	2	3
everX 4m MI	10	0	0
everX 8m MI	9	1	0
everX 4m SN	7	1	2
everX 8m SN	10	0	0

## Data Availability

The original contributions presented in this study are included in the article.
